# The complete mitochondrial genomes of four “bee-killer” assassin bugs (Hemiptera: Reduviidae: *Apiomerus*) recovered using shallow whole-genome sequencing

**DOI:** 10.12688/f1000research.179613.1

**Published:** 2026-06-12

**Authors:** Andrés Gómez-Palacio, Sandra Uribe-Soto, Carolina Ortiz-Muñoz, Dimitri Forero

**Affiliations:** 1 Laboratorio de Investigación en Genética Evolutiva - LIGE., Universidad Pedagogica y Tecnologica de Colombia, Tunja, Boyaca, Colombia; 2 Grupo de Estudios en Genética y Biología Molecular - GEBIMOL, Universidad Pedagógica y Tecnológica de Colombia, Tunja, Boyacá, Colombia; 3 Grupo de Investigación en Sistemática Molecular - GSM, Universidad Nacional de Colombia, Medellín, Colombia; 4 Centro de Investigación La Selva, Corporación Colombiana de Investigación Agropecuaria, AGROSAVIA, Rionegro, Antioquia, Colombia; 5 Universidad Nacional de Colombia, sede Bogotá, Facultad de Ciencias, National University of Colombia Natural Sciences Institute, Bogotá, Bogota, Colombia

**Keywords:** Apiomerus, Apiomerini, mitochondrial genome, mitogenome, Reduviidae, Harpactorinae, Neotropical assassin bugs, genome resource

## Abstract

**Background:**

*Apiomerus* Hahn (“bee-killer” assassin bugs) is a Neotropical genus of Reduviidae with ecological importance as a group of predatory insects and notable morphological diversity. However, complete mitochondrial genome resources have not been available for this genus, limiting its representation in genomic databases and its reuse in comparative studies within Apiomerini and Harpactorinae.

**Methods:**

We generated low-coverage whole-genome sequencing data from four specimens collected in Colombia and assembled and annotated the first complete mitochondrial genomes for *Apiomerus* taxa: *Apiomerus* sp., *A. ochropterus*, *A. luctuosus*, and *A. nitidicollis.* Genome annotation was performed using automated approaches followed by manual curation.

**Results:**

The four mitochondrial genomes ranged from 13.6 to 17.0 kb and contained the typical insect mitochondrial gene complement of 13 protein-coding genes, 22 transfer RNAs, and 2 ribosomal RNAs. All assemblies were AT-rich and showed gene order broadly consistent with other reduviid mitogenomes. In *Apiomerus* sp., some annotations were retained as putative because boundaries for rrnL, rrnS, and trnV could not be resolved with confidence. A phylogenetic tree inferred from concatenated mitochondrial protein-coding genes recovered the four *Apiomerus* taxa as a monophyletic group, in which *A. ochropterus* and *Apiomerus* sp. formed a sister pair, *A. nitidicollis* was sister to that clade, and *A. luctuosus* occupied the basal position among the sampled *Apiomerus* taxa.

**Data summary:**

These assemblies provide the first mitochondrial genome resources for *Apiomerus* and expand the genomic representation of Apiomerini in public databases. The genome sequences, annotations, and accompanying tree resource provide reusable data for future work on comparative mitogenomics, taxonomy, species identification, and phylogenetic studies in Reduviidae.

## Introduction

The genus *Apiomerus* Hahn (Hemiptera: Reduviidae) is one of the most diverse groups of assassin bugs in the Neotropics, comprising more than 100 described species and exhibiting substantial morphological and ecological diversity. ^1, 2^ Species of *Apiomerus* are predatory and contribute to the regulation of other insect populations. ^3^ However, species-level identification within the genus remains challenging because of marked intraspecific chromatic variation and incomplete morphological documentation, which have complicated taxonomic assessment in some groups. ^3, 4^

Mitogenomic data have become an important resource for insect systematics, species identification, and comparative genomic studies. ^5, 6^ Animal mitochondrial genomes are typically conserved in structure and generally contain 37 genes, including 13 protein-coding genes (PCGs), 22 transfer RNAs (tRNAs), and 2 ribosomal RNAs (rRNAs). Compared with single-locus markers such as cox1 or 16S rRNA, complete mitochondrial genomes provide a larger set of homologous characters that can support comparative analyses across taxa. ^7– 9^

Despite the increasing availability of insect mitogenomes, Reduviidae remains sparsely represented in public databases. For *Apiomerus*, complete mitochondrial genome resources have been unavailable, limiting genomic representation of the genus and restricting its inclusion in broader comparative studies within Apiomerini and Harpactorinae. ^2^

Expanding mitogenomic resources for Neotropical reduviids is therefore important for improving taxonomic representation in sequence databases and supporting future systematic, comparative, and biodiversity-oriented research. ^10^ In addition, next-generation sequencing approaches enable recovery of mitochondrial genome data from both recently collected and preserved material, increasing the value of entomological collections as genomic resources. ^10, 11^

Here, we report the first complete mitochondrial genomes for four *Apiomerus* taxa, including three species assigned to the Hirtipes group and one unassigned taxon. These genome assemblies provide the first mitogenomic resource for the genus and expand the genomic representation of *Apiomerus* from Colombia.

## Methods

### Sampling and biological material

Specimens of the assassin bug genus *Apiomerus* Hahn (Hemiptera: Reduviidae) were collected in Colombia between 2018 and 2025 through active daytime manual searches on vegetation in the departments of Antioquia, Casanare, and Santander, at elevations ranging from 184 to 2369 m a.s.l. Geographic coordinates were recorded for all sampling localities.

Morphological identification was based on external diagnostic characters, particularly hemelytral colour patterns, together with examination of male and female genitalia. Species determinations followed the taxonomic treatments and original descriptions of Forero and Weirauch, ^12^ Gil-Santana *et al.,* ^13^ and Carl Stål, ^14, 15^ and were performed by Dr Dimitri Forero.

The ethanol-preserved specimen used for the mitogenome of *Apiomerus ochropterus* (voucher UNAL:MEFLG_Apio093; female) was collected on 13 January 2025 in Reserva Forestal El Centello, Jardín, Antioquia, Colombia (5°30′25.729″N, 75°50′50.262″W; 2369 m; cloud forest), and is deposited in the Francisco Luis Gallego Museum, Universidad Nacional de Colombia.

Additional dry-pinned specimens processed for genomic analyses included: *Apiomerus nitidicollis* (voucher ICN:ICN111295; female), collected on 4 December 2024 in Reserva Natural de la Sociedad Civil San Juan de Tinije, vereda Palmarito, Maní, Casanare (4.8508°N, 72.3719°W; 184 m; gallery forest edge), and deposited in the Instituto de Ciencias Naturales (ICN), Universidad Nacional de Colombia; *Apiomerus luctuosus* (voucher ICN:ICN111161; female), collected on 8 December 2024 in vereda Las Atalayas, hacienda Pajonales, sitio Cunaviche, Aguazul, Casanare (5.0851°N, 72.5040°W; 242 m; piedmont terra firme forest), and deposited in ICN; and *Apiomerus* sp. (voucher IAvH:IAvH-E-206069), collected on 21 February 2018 in vereda La Belleza, Carmen de Chucurí, Santander (6°34′11.5″N, 73°34′12.3″W; 847 m; sandy forest), and deposited in the Instituto Humboldt (IAvH).

Fresh specimens were euthanized by freezing and preserved either dry or in 96% ethanol. For each specimen, one leg was dissected and used for DNA extraction. Voucher specimens are curated in publicly accessible institutional collections, ensuring long-term preservation and traceability of the genomic data.

### DNA extraction, library preparation, and sequencing

Prior to DNA extraction, each dissected leg was briefly surface-cleaned to reduce potential contamination from external tissues and environmental material. Total genomic DNA was extracted from a single leg of each adult specimen using the DNeasy Blood & Tissue Kit (Qiagen, Germany; cat. no. 69504), following the manufacturer’s instructions with minor modifications for insect tissue. Each extraction included 180 μL Buffer ATL and 20 μL Proteinase K, followed by digestion at 56 °C. For *Apiomerus* sp., *A. luctuosus*, and *A. nitidicollis*, the digestion step lasted 3 h, and DNA was eluted in 100 μL Buffer AE. For *A. ochropterus*, digestion was extended to 4 h, and DNA was recovered through two sequential elutions of 25 μL each to maximize yield.

DNA concentration and purity were quantified using a NanoDrop 2000/2000c spectro-photometer (Thermo Fisher Scientific, USA; cat. No. ND-2000) and verified by 1% agarose gel electrophoresis in 1× TAE buffer (Invitrogen, USA; cat. No. 15558–042) with GelRed stain (Biotium, USA; cat. No. 41003). Only samples with concentrations ≥1 ng μL ^−1^ and 260/280 and 230/260 ratios between 1.8 and 2.0 were used for sequencing.

High-quality DNA extracts were selected for shallow whole-genome sequencing (approximately 7× coverage). Paired-end libraries (2 × 150 bp, ~350 bp insert size) were prepared using the NEBNext Ultra II DNA Library Prep Kit for Illumina (New England Biolabs, USA; cat. No. E7645L) according to the manufacturer’s protocol, including size selection with AMPure XP magnetic beads (Beckman Coulter, USA; cat. No. A63881). Library concentrations were determined using a Qubit 4 Fluorometer and the Qubit dsDNA HS Assay Kit (cat. No. Q32854). Libraries were pooled equimolarly and sequenced on an Illumina NovaSeq X platform (Illumina, USA) at Macrogen Inc. (Seoul, South Korea), generating approximately 8–10 Gb of raw paired-end data per sample, corresponding to an estimated genomic coverage of ~7 ×.

### Read processing, mitogenome assembly, and annotation

Raw paired-end Illumina reads were filtered for quality and adapter-trimmed using fastp v0.23.4. ^16^ The resulting high-quality reads were then assembled *de novo* with SPAdes v3.15.5 ^17^ using default parameters optimized for short-read data. Assembled contigs were screened for mitochondrial sequences by BLASTN comparison against the reference mitogenome of *Agriosphodrus dohrni* (Harpactorinae; NCBI Reference Sequence: NC_015842.1).

Initial mitochondrial genome annotation was performed with MITOS2 ^18^ through the Galaxy server. Annotations were subsequently refined by comparison with previously published reduviid mitogenomes and by manual curation of gene boundaries, start and stop codons, and tRNA predictions. Transfer RNA annotations were additionally checked using tRNAscan-SE v2.0.12. ^19^ Circular plots and comparative mitochondrial genome figures were generated in R ^20^ using *circlize* v0.4.17 ^21^ package.

### Phylogenetic analysis

Phylogenetic relationships were inferred using the concatenated amino acid sequences of the 13 mitochondrial protein-coding genes (PCGs: atp6, atp8, cox1–3, cob, nad1–6, and nad4l) from four newly assembled mitogenomes of *Apiomerus* and 22 representative Harpactorinae species retrieved from GenBank. To ensure balanced taxonomic representation and avoid redundancy, a single complete mitochondrial genome accession was selected per species based on BLAST screening and manual curation of available records. The final dataset included representative taxa such as *Epidaus famulus* (NC_085748.1), *Polididus armatissimus* (NC_069625.1), *Rhynocoris altaicus* (PQ613804.1), *R. fuscipes* (MZ440304.1), *Scipinia horrida* (NC_037744.1), *Sclomina erinacea* (ON116856.1), and *S. guangxiensis* (ON116892.1). The Triatominae species *Triatoma infestans* (KY640305.1) was included as outgroup.

Annotated GenBank records were downloaded using NCBI Entrez Direct utilities, and the 13 mitochondrial PCGs were extracted based on annotated coding sequence (CDS) features using a custom Python script. Gene names were standardized across annotations (e.g., cox1/coi, cob/cytb, nad/nd synonyms), and only the first occurrence of each PCG per genome was retained. Amino acid translations were generated from extracted CDS sequences and aligned independently for each gene using MAFFT v7.525 ^22, 23^ under the automatic algorithm selection strategy.

Individual gene alignments were concatenated into a supermatrix using a custom Python script, and a partition file was generated assigning each PCG as an independent data block. The final amino acid matrix comprised 27 taxa (four *Apiomerus* species, 22 Harpactorinae representatives, and one outgroup). Maximum likelihood analyses were conducted in IQ-TREE v2.0.3 ^24^ under partitioned model selection (MFP + MERGE). Branch support was assessed with 1,000 ultrafast bootstrap replicates and 1,000 SH-aLRT replicates.

## Results

### Data quality and preprocessing

Sequencing of the four *Apiomerus* specimens generated between 14.3 million ( *A. nitidicollis*) and 21.1 million ( *A. luctuosus*) raw paired-end reads per sample **(** Table 1 **)**. After quality filtering and adapter trimming, 14.1–20.9 million high-quality reads were retained per sample, corresponding to read retention rates of approximately 97–99%. *A. luctuosus* yielded the largest number of clean reads, whereas *A. nitidicollis* yielded the fewest. These filtered reads were used for *de novo* assembly and mitochondrial genome reconstruction.

**Table 1.** Sequencing output and general assembly statistics for the four *Apiomerus* specimens.

	*A. ochropterus*	*A. nitidicollis*	*A. luctuosus*	*Apiomerus* sp.
Raw	15.981.884	14.308.296	21.075.440	15.889.738
Clean raw	15.812.932	14.146.378	20.875.784	15.732.364
Total length (Mbp)	107.998077	285.625801	388.019607	219.703629
Total contigs	684910	594804	787886	591827
Contigs ≥1 kb	1400	59573	76117	19070
Contigs ≥10 kb	1	21	104	28

### Genome assembly and mitogenome recovery

General *de novo* genome assemblies varied among taxa in total assembled length and contig number **(** Table 1 **)**. Total assembly size ranged from 108.0 Mbp in *A. ochropterus* to 388.0 Mbp in *A. luctuosus*, with intermediate values for *Apiomerus* sp. (219.7 Mbp) and *A. nitidicollis* (285.6 Mbp). The assemblies comprised between 591,827 and 787,886 contigs. The number of contigs ≥1 kb ranged from 1,400 in *A. ochropterus* to 76,117 in *A. luctuosus*, whereas contigs ≥10 kb ranged from 1 to 104. Despite variation in these general assembly statistics, mitochondrial contigs were recovered from all four datasets and used to reconstruct complete mitochondrial genomes for each taxon.

### General features of the mitogenomes and phylogeny

**Figure 1.**
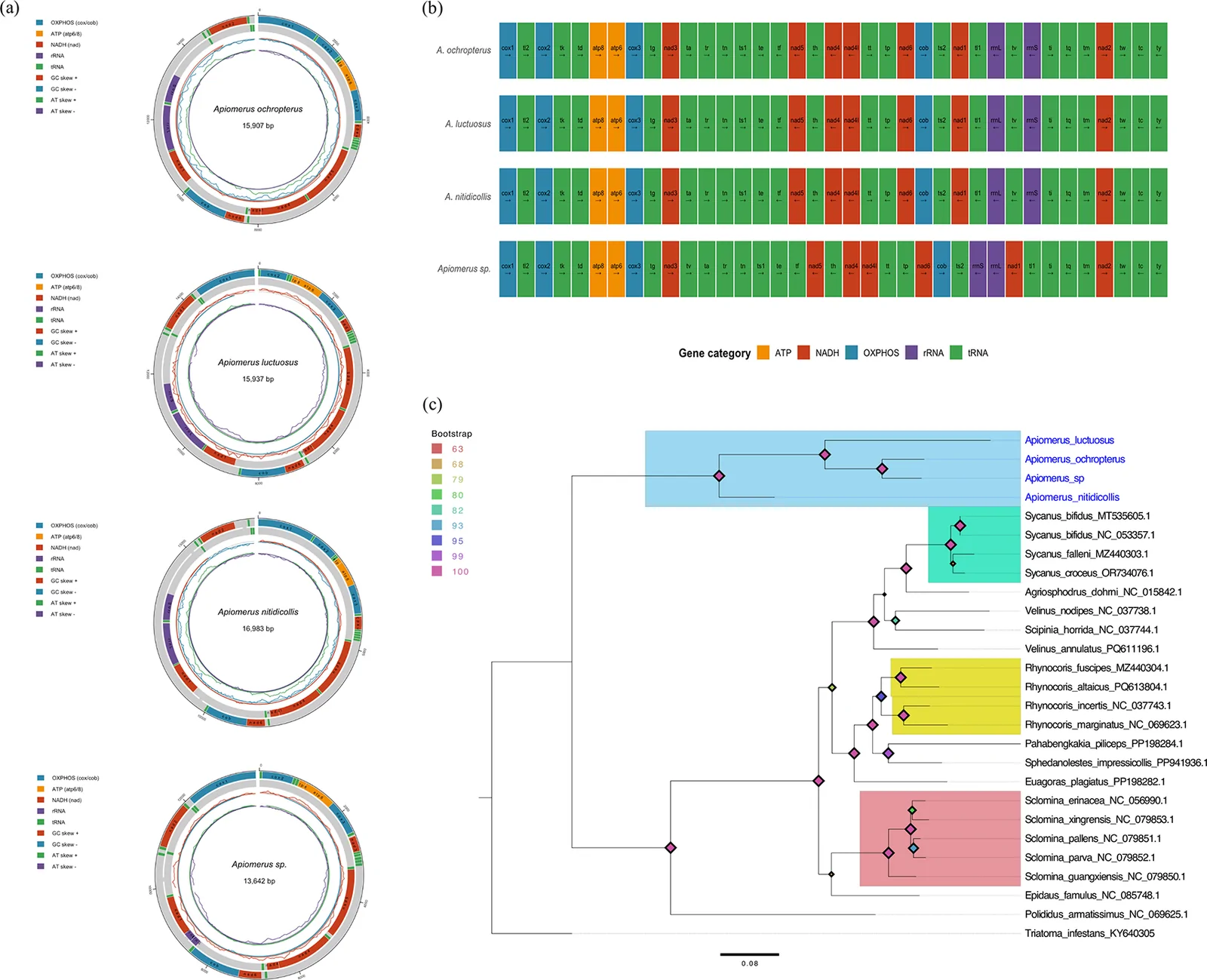
Mitogenome characterization of *Apiomerus.* (a) Circular maps of the complete mitochondrial genomes of *Apiomerus ochropterus*, *A. luctuosus*, *A. nitidicollis*, and *Apiomerus* sp., showing gene annotation and genome organization. (b) Comparative gene order and synteny among the four annotated *Apiomerus* mitogenomes. (c) Maximum-likelihood phylogenetic tree inferred from concatenated mitochondrial protein-coding genes of *Apiomerus* and representative Harpactorinae taxa. *Triatoma infestans* was included as the outgroup. Highlighted boxes indicate recovered genus-level clades. Bootstrap support values from 1,000 replicates are shown at the nodes, and the scale bar indicates amino acid substitutions per site.

Four complete mitochondrial genomes were assembled and annotated for *A. ochropterus*, *A. nitidicollis*, *A. luctuosus*, and *Apiomerus* sp. **(** Figure 1a; Table 2). The assembled mitogenomes measured 15,907 bp in *A. ochropterus*, 16,983 bp in *A. nitidicollis*, 15,937 bp in *A. luctuosus*, and 13,642 bp in *Apiomerus* sp.; all were AT-rich. Three mitogenomes showed the standard insect mitochondrial gene complement of 13 protein-coding genes, 22 transfer RNAs, 2 ribosomal RNAs, and a control region, whereas the *Apiomerus* sp. assembly should be interpreted cautiously because annotation of the canonical trnV and rrnL/rrnS boundaries was uncertain. An alternative trnV candidate was predicted elsewhere in the mitogenome, but its weak structural support and overlap with adjacent features suggest that it is more likely to represent an annotation artefact than a genuine rearrangement.

**Table 2.** General features of the assembled mitochondrial genomes of four *Apiomerus* taxa.

	*A. ochropterus*	*A. nitidicollis*	*A. luctuosus*	*Apiomerus* sp.
Mitogenome length (bp)	15.907	16.983	15.937	13.642
A + T (%)	72.17	71.76	70.7	68.61
PCGs	13	13	13	13
tRNAs	22	22	22	22
rRNAs	2	2	2	2
Control region	1	1	1	1

The four annotated mitogenomes showed broadly conserved organization and clear synteny, with no major changes in the relative order of protein-coding genes, rRNAs, or most tRNAs **(** Figure 1b **)**. Differences among taxa were limited mainly to local variation in intergenic spacing and in the annotation of a small number of features, particularly in *Apiomerus* sp. near the rrnL-rrnS region. The comparative panel in Figure 1b supports overall structural conservation across the four taxa.

Phylogenetic relationships inferred from concatenated mitochondrial PCGs recovered *Apiomerus* as a monophyletic group **(** Figure 1c **)**. Within the genus, *A. ochropterus* and *Apiomerus* sp. were recovered as sister taxa, *A. luctuosus* grouped with that pair, and *A. nitidicollis* was sister to the remaining sampled congeners. The broader tree also recovered the other highlighted harpactorine genus-level clades shown in Figure 1c. Bootstrap values were generally high, although support for the *A. ochropterus* + *Apiomerus* sp. node was moderate.

## Ethical considerations

This study involved the collection of non-commercial, non-endangered insect specimens ( *Apiomerus*, Hemiptera: Reduviidae) for taxonomic and genomic research purposes. Field sampling was conducted in Colombia under official collection permits Resolución No. 0147 (2023) issued by the Ministerio de Ambiente y Desarrollo Sostenible and Resoluciones No. 0255 (2014) and No. 000697 (2025) issued by the Autoridad Nacional de Licencias Ambientales (ANLA) to the Universidad Nacional de Colombia. All sampling procedures complied with national biodiversity regulations and institutional guidelines. No protected vertebrate species, human subjects, or regulated experimental animals were involved in this research, and no additional ethical approval was required. The authors declare no conflicts of interest related to specimen collection or research activities.

## Data Availability

The underlying data for this article is available from the National Center for Biotechnology Information (NCBI) repositories, including NCBI BioProject, NCBI Sequence Read Archive (SRA), and NCBI GenBank/Nucleotide. NCBI BioProject: Raw Illumina sequencing data and mitochondrial genome assemblies for “The complete mitochondrial genomes of four ‘bee-killer’ assassin bugs (Hemiptera: Reduviidae: *Apiomerus*) recovered using shallow whole-genome sequencing”. BioProject accession: PRJNA1443192. Direct repository link: https://www.ncbi.nlm.nih.gov/bioproject/PRJNA1443192 The project contains the following underlying data:
• Raw Illumina paired-end sequencing reads for *Apiomerus ochropterus*: NCBI SRA accession SRR38001471.
https://www.ncbi.nlm.nih.gov/sra/SRR38001471
• Raw Illumina paired-end sequencing reads for *Apiomerus nitidicollis*: NCBI SRA accession SRR38001472.
https://www.ncbi.nlm.nih.gov/sra/SRR38001472
• Raw Illumina paired-end sequencing reads for *Apiomerus luctuosus*: NCBI SRA accession SRR38001473.
https://www.ncbi.nlm.nih.gov/sra/SRR38001473
• Raw Illumina paired-end sequencing reads for *Apiomerus* sp.: NCBI SRA accession SRR38001470.
https://www.ncbi.nlm.nih.gov/sra/SRR38001470
• Complete mitochondrial genome assembly of *Apiomerus ochropterus*: NCBI GenBank accession PZ457773.
https://www.ncbi.nlm.nih.gov/nuccore/PZ457773
• Complete mitochondrial genome assembly of *Apiomerus nitidicollis*: NCBI GenBank accession PZ457772.
https://www.ncbi.nlm.nih.gov/nuccore/PZ457772
• Complete mitochondrial genome assembly of *Apiomerus luctuosus*: NCBI GenBank accession PZ457771.
https://www.ncbi.nlm.nih.gov/nuccore/PZ457771
• Complete mitochondrial genome assembly of *Apiomerus* sp.: NCBI GenBank accession PZ255051.
https://www.ncbi.nlm.nih.gov/nuccore/PZ255051 No extended data are associated with this article.
