# Mitochondrial Genome Assemblies of Four “Bee-Assassin” (Hemiptera: Reduviidae: *Apiomerus*) Recovered Using Shallow Whole-Genome sequencing

**DOI:** 10.12688/f1000research.179613.2

**Published:** 2026-08-26

**Authors:** Andrés Gómez-Palacio, Sandra Uribe-Soto, Carolina Ortiz-Muñoz, Dimitri Forero

**Affiliations:** 1 Laboratorio de Investigación en Genética Evolutiva - LIGE., Universidad Pedagogica y Tecnologica de Colombia, Tunja, Boyaca, Colombia; 2 Grupo de Estudios en Genética y Biología Molecular - GEBIMOL, Universidad Pedagógica y Tecnológica de Colombia, Tunja, Boyacá, Colombia; 3 Grupo de Investigación en Sistemática Molecular - GSM, Universidad Nacional de Colombia, Medellín, Colombia; 4 Centro de Investigación La Selva, Corporación Colombiana de Investigación Agropecuaria, AGROSAVIA, Rionegro, Antioquia, Colombia; 5 Universidad Nacional de Colombia, sede Bogotá, Facultad de Ciencias, National University of Colombia Natural Sciences Institute, Bogotá, Bogota, Colombia

**Keywords:** Apiomerus, Apiomerini, mitochondrial genome, mitogenome, Reduviidae, Harpactorinae, Neotropical assassin bugs, genome resource

## Abstract

*Apiomerus* (“bee-assassin” assassin bugs) is a Neotropical genus of Reduviidae with ecological importance as a group of predatory insects and notable morphological diversity, yet mitochondrial genomic resources have remained unavailable for the genus. We generated low-coverage whole-genome sequencing data from four vouchered specimens collected in Colombia and reconstructed mitochondrial genomes for *Apiomerus* sp., *A. ochropterus*, *A. luctuosus*, and *A. nitidicollis*. Quality-filtered reads were assembled using an organelle-specific GetOrganelle workflow, and the resulting assembly graphs and candidate sequences were evaluated against initial SPAdes/BLASTN-based reconstructions. GetOrganelle recovered complete circular mitochondrial genomes for *Apiomerus* sp., *A. ochropterus*, and *A. nitidicollis*, whereas *A. luctuosus* was represented by a single gap-free, non-circularized mitochondrial scaffold in which the control region was not completely recovered. The selected assemblies ranged from 14,943 to 19,405 bp and were strongly AT-rich. Annotation and manual curation recovered the complete complement of 13 protein-coding genes, 22 tRNA genes, and two rRNA genes in all four taxa. Comparative analysis showed complete conservation of mitochondrial gene content, gene order, and transcriptional orientation, with no evidence of gene rearrangements among the sampled species. Phylogenetic analysis based on concatenated mitochondrial protein-coding genes recovered the four *Apiomerus* taxa as a monophyletic group, with *A. ochropterus* and *Apiomerus* sp. forming a sister pair, *A. nitidicollis* sister to that clade, and *A. luctuosus* occupying the basal position among the sampled taxa. These assemblies constitute the first mitochondrial genomic resources for *Apiomerus* and expand the representation of Apiomerini for comparative mitogenomic, taxonomic, and phylogenetic studies.

## Introduction

The genus *Apiomerus* Hahn (Hemiptera: Reduviidae) is one of the most diverse groups of assassin bugs in the Neotropics, comprising more than 100 described species and exhibiting substantial morphological and ecological diversity. ^1, 2^ Species of *Apiomerus* are predatory and contribute to the regulation of other insect populations. ^3^ However, species-level identification within the genus remains challenging because of marked intraspecific chromatic variation and incomplete morphological documentation, which have complicated taxonomic assessment in some groups. ^3, 4^

Mitogenomic data have become an important resource for insect systematics, species identification, and comparative genomic studies. ^5, 6^ Animal mitochondrial genomes are typically conserved in structure and generally contain 37 genes, including 13 protein-coding genes (PCGs), 22 transfer RNAs (tRNAs), and 2 ribosomal RNAs (rRNAs). Compared with single-locus markers such as cytochrome c oxidase subunit I (COI; cox1) or 16S rRNA, complete mitochondrial genomes provide a larger set of homologous characters that can support comparative analyses across taxa. ^7– 9^

Despite the increasing availability of insect mitogenomes, Reduviidae remains sparsely represented in public databases. For *Apiomerus*, complete mitochondrial genome resources have been unavailable, limiting genomic representation of the genus and restricting its inclusion in broader comparative studies within Apiomerini and Harpactorinae. ^2^

Expanding mitogenomic resources for Neotropical reduviids is therefore important for improving taxonomic representation in sequence databases and supporting future systematic, comparative, and biodiversity-oriented research. ^10^ In addition, next-generation sequencing approaches enable recovery of mitochondrial genome data from both recently collected and preserved material, increasing the value of entomological collections as genomic resources. ^10, 11^

Here, we report the first complete mitochondrial genomes for four *Apiomerus* taxa, including three species assigned to the Hirtipes group and one unassigned taxon. These genome assemblies provide the first mitogenomic resource for the genus and expand the genomic representation of *Apiomerus* from Colombia.

## Methods

### Sampling and biological material

Specimens of the assassin bug genus *Apiomerus* Hahn (Hemiptera: Reduviidae) were collected in Colombia between 2018 and 2025 through active daytime manual searches on vegetation in the departments of Antioquia, Casanare, and Santander, at elevations ranging from 184 to 2369 m a.s.l. Geographic coordinates were recorded for all sampling localities.

Morphological identification was based on external diagnostic characters, particularly hemelytral colour patterns, together with examination of male and female genitalia. Species determinations followed the taxonomic treatments and original descriptions of Forero and Weirauch, ^12^ Gil-Santana *et al.,* ^13^ and Carl Stål, ^14, 15^ and were performed by Dr Dimitri Forero.

The ethanol-preserved specimen used for the mitogenome of *Apiomerus ochropterus* (voucher UNAL:MEFLG_Apio093; female) was collected on 13 January 2025 in Reserva Forestal El Centello, Jardín, Antioquia, Colombia (5°30′25.729″N, 75°50′50.262″W; 2369 m; cloud forest), and is deposited in the Francisco Luis Gallego Museum, Universidad Nacional de Colombia.

Additional dry-pinned specimens processed for genomic analyses included: *Apiomerus nitidicollis* (voucher ICN:ICN111295; female), collected on 4 December 2024 in Reserva Natural de la Sociedad Civil San Juan de Tinije, vereda Palmarito, Maní, Casanare (4.8508°N, 72.3719°W; 184 m; gallery forest edge), and deposited in the Instituto de Ciencias Naturales (ICN), Universidad Nacional de Colombia; *Apiomerus luctuosus* (voucher ICN:ICN111161; female), collected on 8 December 2024 in vereda Las Atalayas, hacienda Pajonales, sitio Cunaviche, Aguazul, Casanare (5.0851°N, 72.5040°W; 242 m; piedmont terra firme forest), and deposited in ICN; and *Apiomerus* sp. (voucher IAvH:IAvH-E-206069), collected on 21 February 2018 in vereda La Belleza, Carmen de Chucurí, Santander (6°34′11.5″N, 73°34′12.3″W; 847 m; sandy forest), and deposited in the Instituto Humboldt (IAvH).

Fresh specimens were euthanized by freezing and preserved either dry or in 96% ethanol. For each specimen, one leg was dissected and used for DNA extraction. Voucher specimens are curated in publicly accessible institutional collections, ensuring long-term preservation and traceability of the genomic data.

### DNA extraction, library preparation, and sequencing

Prior to DNA extraction, each dissected leg was briefly surface-cleaned to reduce potential contamination from external tissues and environmental material. Total genomic DNA was extracted from a single leg of each adult specimen using the DNeasy Blood & Tissue Kit (Qiagen, Germany; cat. no. 69504), following the manufacturer’s instructions with minor modifications for insect tissue. Each extraction included 180 μL Buffer ATL and 20 μL Proteinase K, followed by digestion at 56 °C. For *Apiomerus* sp., *A. luctuosus*, and *A. nitidicollis*, the digestion step lasted 3 h, and DNA was eluted in 100 μL Buffer AE. For *A. ochropterus*, digestion was extended to 4 h, and DNA was recovered through two sequential elutions of 25 μL each to maximize yield.

DNA concentration and purity were quantified using a NanoDrop 2000/2000c spectro-photometer (Thermo Fisher Scientific, USA; cat. No. ND-2000) and verified by 1% agarose gel electrophoresis in 1× TAE buffer (Invitrogen, USA; cat. No. 15558–042) with GelRed stain (Biotium, USA; cat. No. 41003). Only samples with concentrations ≥1 ng μL ^−1^ and 260/280 and 260/230 ratios between 1.8 and 2.0 were used for sequencing.

High-quality DNA extracts were selected for shallow whole-genome sequencing (approximately 7× coverage). Paired-end libraries (2 × 150 bp, ~350 bp insert size) were prepared using the NEBNext Ultra II DNA Library Prep Kit for Illumina (New England Biolabs, USA; cat. No. E7645L) according to the manufacturer’s protocol, including size selection with AMPure XP magnetic beads (Beckman Coulter, USA; cat. No. A63881). Library concentrations were determined using a Qubit 4 Fluorometer and the Qubit dsDNA HS Assay Kit (cat. No. Q32854). Libraries were pooled equimolarly and sequenced on an Illumina NovaSeq X platform (Illumina, USA) at Macrogen Inc. (Seoul, South Korea), generating approximately 8–10 Gb of raw paired-end data per sample, corresponding to an estimated genomic coverage of ~7 ×.

### Read processing, mitogenome assembly, and annotation

Raw paired-end Illumina reads were quality-filtered and adapter-trimmed using fastp v0.23.4. ^16^ The resulting reads were initially assembled *de novo* with SPAdes v3.15.5 ^17^ using default parameters for short-read data. Contigs of putative mitochondrial origin were identified by BLASTN comparison against the reference mitogenome of *Agriosphodrus dohrni* (Harpactorinae; NCBI Reference Sequence NC_015842.1). These SPAdes-derived mitochondrial reconstructions were retained as an independent assembly for comparison with the organelle-specific assemblies described below.

The filtered paired-end reads were subsequently reassembled using GetOrganelle v1.7.7.1 ^18^ in animal mitochondrial mode (-F animal_mt), with the A. dohrni mitogenome supplied as the seed sequence. Assemblies were generated using k-mer sizes of 21, 45, 65, 85, and 105. Sample-specific word sizes and extension-round limits were used to accommodate differences in mitochondrial read coverage and assembly-graph complexity: a word size of 80 and a maximum of 30 extension rounds were used for *A. ochropterus*; a word size of 100 and 10 extension rounds for *A. nitidicollis*; a word size of 90 and 20 extension rounds for *A. luctuosus*; and an automatically estimated word size and 10 extension rounds for *Apiomerus* sp. Assembly graphs were examined to distinguish closed circular paths from unresolved or linear scaffolds.

The selected GetOrganelle assemblies were compared reciprocally with the original SPAdes/BLASTN-derived mitochondrial reconstructions using BLASTN. Comparisons included nucleotide identity, query coverage, alignment continuity, total assembly length, and the presence of duplicated, additional, or unresolved terminal regions.

The original filtered paired-end reads were independently mapped to the SPAdes/BLASTN-derived and GetOrganelle assemblies using Minimap2 v2.28-r1209. ^19^ Alignment files were processed using SAMtools v1.18, ^20^ and assembly support was evaluated from coverage breadth at ≥1× and ≥10×, read-depth distributions, depth uniformity, and the number of properly paired reads. For each putatively circular GetOrganelle assembly, reads were additionally mapped across a reconstructed circular-junction reference to test whether coverage and paired-read support continued uninterrupted across the sequence boundary. The *A. luctuosus* assembly was retained as a non-circular scaffold and was therefore not subjected to circular-junction validation. An assembly was classified as circular only when GetOrganelle recovered a closed path without ambiguous bases and the reconstructed junction was supported by continuous read coverage and properly paired reads.

Initial mitochondrial genome annotation was performed with MITOS2 ^21^ through the Galaxy server. Annotations were subsequently refined by comparison with previously published reduviid mitogenomes and by manual curation of gene boundaries, start and stop codons, and tRNA predictions. Transfer RNA annotations were independently evaluated using tRNAscan-SE v2.0.12 ^22^ in organellar mode, and discrepant predictions were resolved through comparison of genomic coordinates, anticodons, predicted secondary structures, and conserved mitochondrial gene order. Circular mitochondrial genome plots and comparative figures were generated in R ^23^ using circlize v0.4.17 ^24^ package.

### Phylogenetic analysis

Phylogenetic relationships were inferred using the concatenated amino acid sequences of the 13 mitochondrial protein-coding genes (PCGs: atp6, atp8, cox1–3, cob, nad1–6, and nad4l) from four newly assembled mitogenomes of *Apiomerus* and 22 representative Harpactorinae species retrieved from GenBank. To ensure balanced taxonomic representation and avoid redundancy, a single complete mitochondrial genome accession was selected per species based on BLAST screening and manual curation of available records. The final dataset included representative taxa such as *Epidaus famulus* (NC_085748.1), *Polididus armatissimus* (NC_069625.1), *Rhynocoris altaicus* (PQ613804.1), *R. fuscipes* (MZ440304.1), *Scipinia horrida* (NC_037744.1), *Sclomina erinacea* (ON116856.1), and *S. guangxiensis* (ON116892.1). The Triatominae species *Triatoma infestans* (KY640305.1) was included as outgroup.

Annotated GenBank records were downloaded using NCBI Entrez Direct utilities, and the 13 mitochondrial PCGs were extracted based on annotated coding sequence (CDS) features using a custom Python script. Gene names were standardized across annotations (e.g., cox1/coi, cob/cytb, nad/nd synonyms), and only the first occurrence of each PCG per genome was retained. Amino acid translations were generated from extracted CDS sequences and aligned independently for each gene using MAFFT v7.525 ^25, 26^ under the automatic algorithm selection strategy.

Individual gene alignments were concatenated into a supermatrix using a custom Python script, and a partition file was generated assigning each PCG as an independent data block. The final amino acid matrix comprised 27 taxa (four *Apiomerus* species, 22 Harpactorinae representatives, and one outgroup). Maximum likelihood analyses were conducted in IQ-TREE v2.0.3 ^27^ under partitioned model selection (MFP+MERGE). Branch support was assessed with 1,000 ultrafast bootstrap replicates and 1,000 SH-aLRT replicates.

## Results

### Data quality and preprocessing

Sequencing generated 14.3–21.1 million raw paired-end reads per specimen ( Table 1). After quality filtering and adapter trimming, 14.1–20.9 million reads were retained, corresponding to retention rates of 98.87–99.05%. *A. luctuosus* yielded the largest number of clean reads, whereas *A. nitidicollis* yielded the fewest. The filtered reads were subsequently used for SPAdes-based *de novo* assembly and GetOrganelle mitochondrial genome reconstruction.

**Table 1.** Read-processing and SPAdes-based whole-genome assembly statistics for the four *Apiomerus* specimens.

	*A. ochropterus*	*A. nitidicollis*	*A. luctuosus*	*Apiomerus* sp.
Raw reads, R1 + R2	15,981,884	14,308,296	21,075,440	15,889,738
Clean reads, R1 + R2	15,812,932	14,146,378	20,875,784	15,732,364
Reads retained (%)	98.95	98.87	99.05	99.01
SPAdes assembly size (Mbp)	108	285.63	388.02	219.7
Total SPAdes contigs	684,910	594,804	787,886	591,827
Contigs ≥1 kb	1,400	59,573	76,117	19,070
Contigs ≥10 kb	1	21	104	28
N50 (bp)	241	666	654	389
Longest contig (bp)	15,907	20,060	45,874	19,695

### Assembly comparison and read-based validation

**Figure 1.**
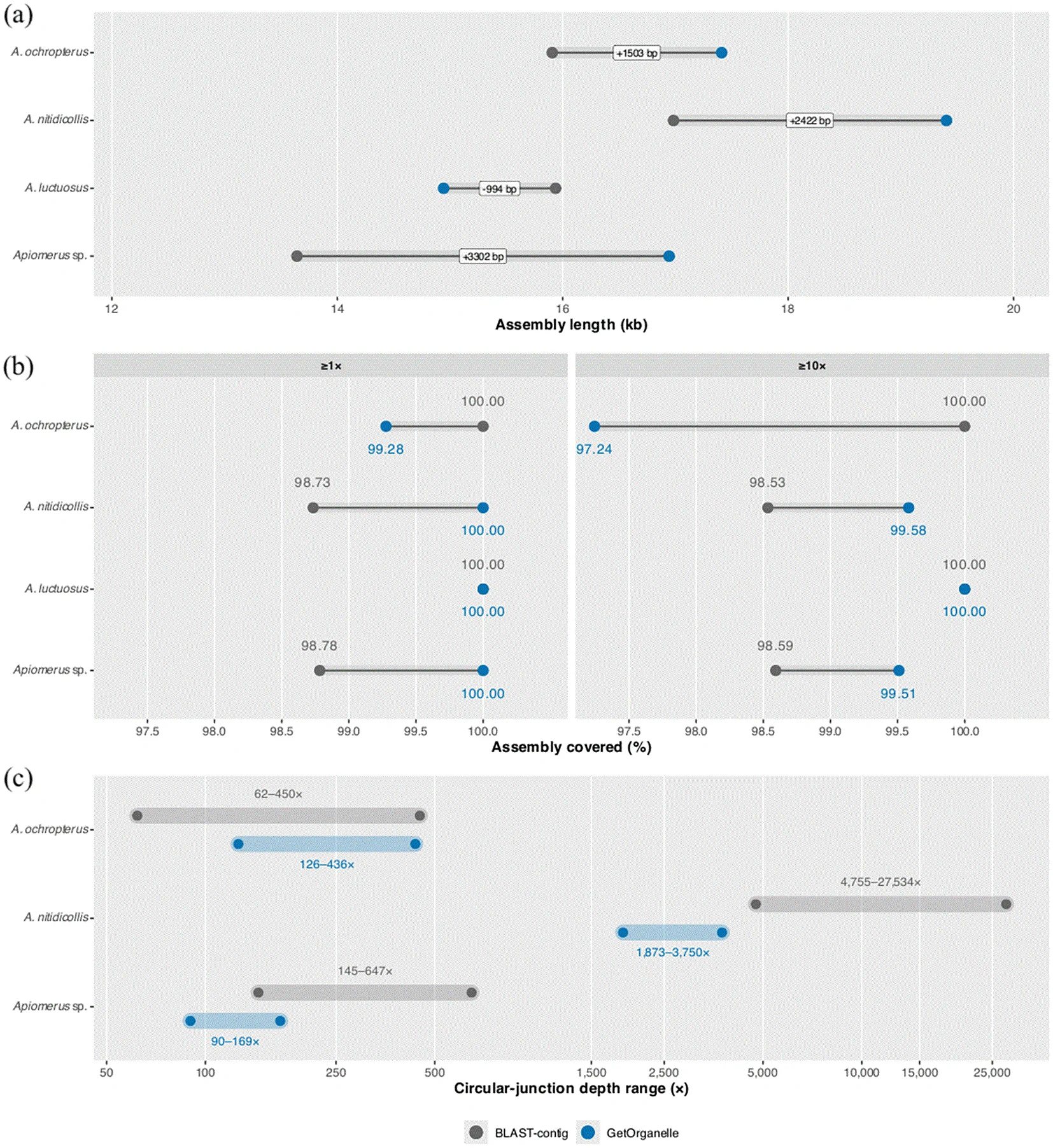
Comparison and read-based validation of the SPAdes/BLAST-contig and GetOrganelle mitochondrial assemblies. (a) Assembly lengths and length differences. (b) Percentage of each assembly covered at ≥1× and ≥10×. (c) Read-depth ranges across reconstructed circular junctions. Grey and blue indicate BLAST-contig and GetOrganelle assemblies, respectively. *A. luctuosus* was excluded from panel (c) because its assembly remained non-circularized.

Reciprocal whole-sequence comparisons showed that the shared regions of the original SPAdes/BLASTN-derived reconstructions and the GetOrganelle assemblies had 95.02–99.97% nucleotide identity. Relative to the earlier sequences, the GetOrganelle assemblies were 1,503 bp longer for *A. ochropterus*, 2,422 bp longer for *A. nitidicollis*, and 3,302 bp longer for *Apiomerus* sp., whereas the *A. luctuosus* scaffold was 994 bp shorter ( Figure 1a). The GetOrganelle assemblies contained 93.76–100% of the earlier sequences, whereas the earlier reconstructions represented only 80.65–100% of the GetOrganelle assemblies. Most differences therefore involved terminal, control-region, or previously unresolved portions rather than extensive divergence across the shared mitochondrial sequence.

Read mapping supported the selected GetOrganelle assemblies. Coverage breadth ranged from 99.28% to 100% at ≥1× and from 97.24% to 100% at ≥10× ( Figure 1b). Read depth was more uniform in the GetOrganelle assemblies of *A. nitidicollis*, *A. luctuosus*, and *Apiomerus* sp., whereas *A. ochropterus* showed a modest increase in depth variability. Properly paired-read support increased for the GetOrganelle assemblies of *A. ochropterus*, *A. nitidicollis*, and *Apiomerus* sp., but decreased for the shorter *A. luctuosus* scaffold.

The three circular assemblies showed uninterrupted read coverage across their reconstructed junctions, with no zero-coverage positions within the evaluated windows ( Figure 1c). Mean junction depths were 250.9× for *A. ochropterus*, 2,816.0× for *A. nitidicollis*, and 136.8× for *Apiomerus* sp. The corresponding junctions were supported by 31, 1,506, and 77 properly paired reads, respectively. Because the *A. luctuosus* assembly remained non-circularized, it was evaluated through terminal-coverage analysis rather than circular-junction validation.

### General features and organization of the mitochondrial genomes

**Figure 2.**
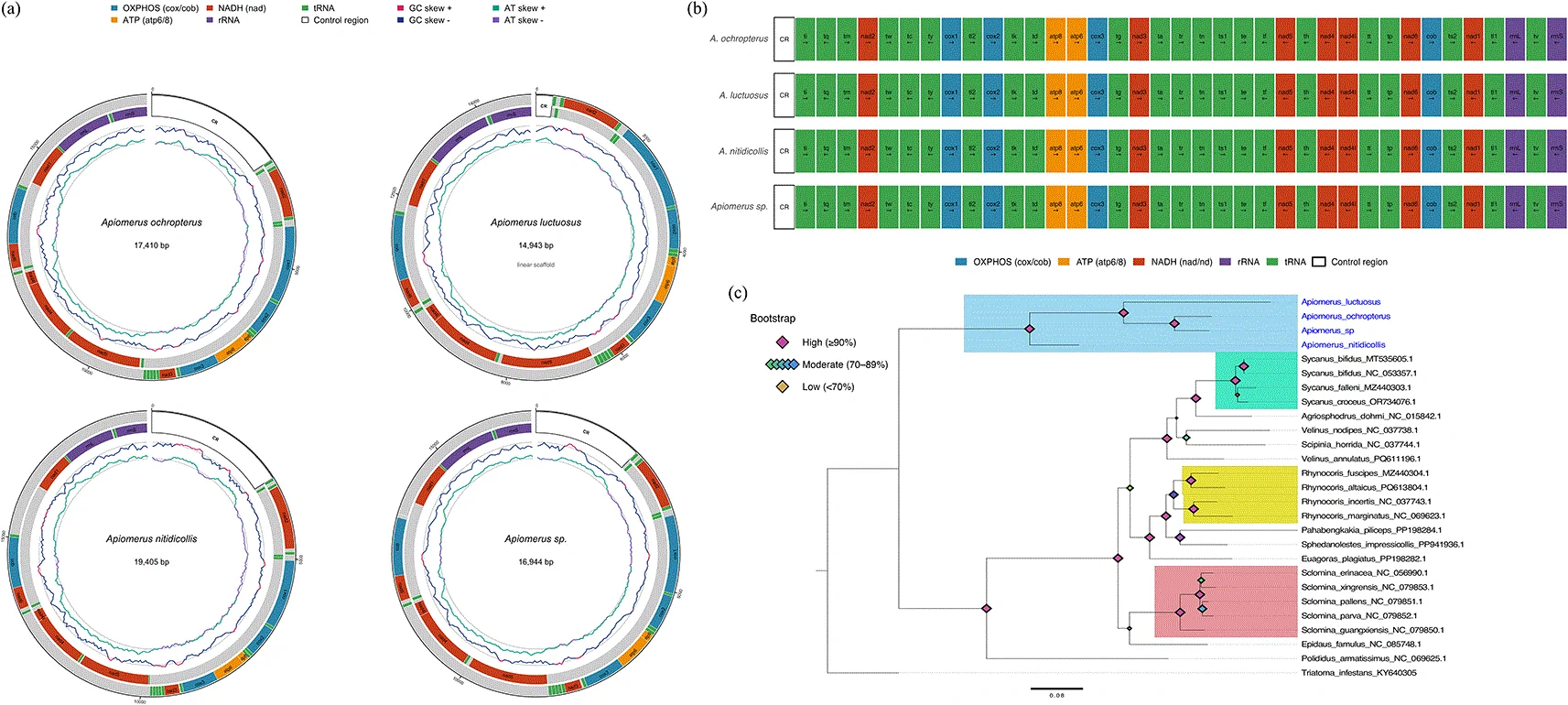
Mitochondrial genome organization, synteny, and phylogeny of *Apiomerus*. (a) Circular maps of the four selected mitochondrial assemblies, showing gene annotations, control regions, and GC/AT skews. The *A. luctuosus* scaffold is displayed circularly for comparison. (b) Conserved mitochondrial gene order and transcriptional orientation. (c) Maximum-likelihood phylogeny based on concatenated mitochondrial protein-coding genes, with *Triatoma infestans* as the outgroup. Node colors indicate bootstrap support from 1,000 replicates.

The GetOrganelle assemblies selected after comparative and read-based validation measured 17,410 bp in *A. ochropterus*, 19,405 bp in *A. nitidicollis*, 14,943 bp in *A. luctuosus*, and 16,944 bp in *Apiomerus* sp., with A+T contents of 72.36%, 73.27%, 70.62%, and 70.25%, respectively ( Table 2; Figure 2a). Complete circular mitogenomes were recovered for *A. ochropterus*, *A. nitidicollis*, and *Apiomerus* sp., whereas *A. luctuosus* was represented by a non-circularized scaffold in which the control region was incompletely recovered. Nevertheless, all four assemblies contained 13 protein-coding genes, 22 tRNA genes, and two rRNA genes.

**Table 2.** Main features of the selected GetOrganelle mitochondrial assemblies.

	*A. ochropterus*	*A. nitidicollis*	*A. luctuosus*	*Apiomerus* sp.
Assembly length (bp)	17,410	19,405	14,943	16,944
A+T content (%)	72.36	73.27	70.62	70.25
Protein-coding genes	13	13	13	13
tRNA genes	22	22	22	22
rRNA genes	2	2	2	2
Control-region recovery	Complete	Complete	Incomplete	Complete
Assembly structure	Circular	Circular	Linear scaffold	Circular
Ambiguous bases	0	0	0	0

After annotation refinement and manual curation, the four assemblies showed complete conservation of mitochondrial gene content, gene order, and transcriptional orientation, with no detected rearrangements of protein-coding genes, rRNAs, or tRNAs ( Figure 2b). Differences among the assemblies were therefore associated primarily with genome length, control-region recovery, and intergenic spacing rather than with changes in mitochondrial gene organization.

Phylogenetic relationships inferred from concatenated mitochondrial PCGs recovered *Apiomerus* as a monophyletic group ( Figure 2c). Within the genus, *A. ochropterus* and *Apiomerus* sp. were recovered as sister taxa, *A. nitidicollis* was sister to that clade, and *A. luctuosus* occupied the basal position among the sampled *Apiomerus* taxa. The broader tree also recovered the other highlighted harpactorine genus-level clades shown in Figure 2c. Bootstrap values were generally high, although support for the *A. ochropterus* + *Apiomerus* sp. node was moderate.

## Ethical considerations

This study involved the collection of non-commercial, non-endangered insect specimens ( *Apiomerus*, Hemiptera: Reduviidae) for taxonomic and genomic research purposes. Field sampling was conducted in Colombia under official collection permits Resolución No. 0147 (2023) issued by the Ministerio de Ambiente y Desarrollo Sostenible and Resoluciones No. 0255 (2014) and No. 000697 (2025) issued by the Autoridad Nacional de Licencias Ambientales (ANLA) to the Universidad Nacional de Colombia. All sampling procedures complied with national biodiversity regulations and institutional guidelines. No protected vertebrate species, human subjects, or regulated experimental animals were involved in this research, and no additional ethical approval was required. The authors declare no conflicts of interest related to specimen collection or research activities.

## Data Availability

The underlying data for this article is available from the National Center for Biotechnology Information (NCBI) repositories, including NCBI BioProject, NCBI Sequence Read Archive (SRA), and NCBI GenBank/Nucleotide. NCBI BioProject: Raw Illumina sequencing data and mitochondrial genome assemblies for “The complete mitochondrial genomes of four ‘bee-assassin’ assassin bugs (Hemiptera: Reduviidae: *Apiomerus*) recovered using shallow whole-genome sequencing”. BioProject accession: PRJNA1443192. Direct repository link: https://www.ncbi.nlm.nih.gov/bioproject/PRJNA1443192 The project contains the following underlying data:
• Raw Illumina paired-end sequencing reads for *Apiomerus ochropterus*: NCBI SRA accession SRR38001471.
https://www.ncbi.nlm.nih.gov/sra/SRR38001471
• Raw Illumina paired-end sequencing reads for *Apiomerus nitidicollis*: NCBI SRA accession SRR38001472.
https://www.ncbi.nlm.nih.gov/sra/SRR38001472
• Raw Illumina paired-end sequencing reads for *Apiomerus luctuosus*: NCBI SRA accession SRR38001473.
https://www.ncbi.nlm.nih.gov/sra/SRR38001473
• Raw Illumina paired-end sequencing reads for *Apiomerus* sp.: NCBI SRA accession SRR38001470.
https://www.ncbi.nlm.nih.gov/sra/SRR38001470
• Mitochondrial genome assembly of *Apiomerus ochropterus*: NCBI GenBank accession PZ457773.
https://www.ncbi.nlm.nih.gov/nuccore/PZ457773
• Mitochondrial genome assembly of *Apiomerus nitidicollis*: NCBI GenBank accession PZ457772.
https://www.ncbi.nlm.nih.gov/nuccore/PZ457772
• Mitochondrial genome assembly of *Apiomerus luctuosus*: NCBI GenBank accession PZ457771.
https://www.ncbi.nlm.nih.gov/nuccore/PZ457771
• Mitochondrial genome assembly of *Apiomerus* sp.: NCBI GenBank accession PZ255051.
https://www.ncbi.nlm.nih.gov/nuccore/PZ255051 No extended data are associated with this article.
