# Supplementary material for: Mitochondrial Genome Assemblies of Four “Bee-Assassin” (Hemiptera: Reduviidae: Apiomerus) Recovered Using Shallow Whole-Genome sequencing
Source: F1000Res. 2026 Aug 26;15:921. Originally published 2026 Jun 12. [Version 2] doi: 10.12688/f1000research.179613.2 (PMC13594599; doi:10.12688/f1000research.179613.2)
Supplement: Peer review history

# Reviewer report: Reviewer response for version 2

**DOI:** 10.5256/f1000research.208114.r518524

**Published:** 2026-09-28

**Authors:** Kanishka Senevirathna

**Affiliations:** 1 Department of Plant Science, University of Saskatchewan College of Agriculture and Bioresources (Ringgold ID: 98627), Saskatoon, Saskatchewan, Canada; 2 University of Alberta, Edmonton, Canada

This is a solid, well-documented genomic resource paper, the sequencing, assembly, and validation workflow is unusually rigorous for what is essentially a first-report/data-note style manuscript (dual-assembly comparison, read-mapping validation, junction-support testing). However, there are several methodological, interpretive, and presentation issues that should be addressed before indexing.

 1. The specimen collection details (voucher numbers, sex, preservation method, dates, localities, coordinates, elevation, habitat, and repository) are currently presented as a single continuous paragraph in the Methods (“Sampling and biological material”). This information is dense, non-uniform in structure across the four taxa, and difficult to scan or extract for reuse (e.g., by researchers wishing to cite voucher numbers or coordinates in later comparative work).

 I suggest presenting these data as a table. This would let readers immediately see, for example, that the four specimens span very different collection dates (2018 vs. 2024–2025) and preservation methods (one ethanol-preserved, three dry-pinned), both factors that plausibly bear on DNA yield and quality, and which are otherwise easy to miss when reading the prose version.

 2. Could the authors briefly explain why A. ochropterus needed a longer digestion and two elutions? Was this because of low DNA yield or the sample condition?

 Also, since three of the four specimens were dry-pinned, it would be helpful to report the actual DNA concentrations and purity ratios (260/280 and 260/230) for each sample in Table 1, rather than just the quality thresholds. This would help readers assess DNA quality and understand whether it may have contributed to the incomplete A. luctuosus assembly.

 3. Every conclusion (genome length, AT content, gene order conservation, phylogenetic placement) rests on a single specimen per species. This is standard practice for "first mitogenome" reports, but the manuscript should explicitly acknowledge this as a limitation

 4. The authors have taken some steps to validate the mitogenome assemblies, but it would be helpful to explain how they ruled out NUMTs (nuclear copies of mitochondrial DNA). This is especially important for A. luctuosus, where the assembly was not circularized. A brief explanation or discussion of this limitation would strengthen the manuscript.

 5. The manuscript would benefit from a short Discussion section to explain the main findings. The authors could compare the genome sizes and AT content with other Harpactorinae, discuss the phylogenetic results, and explain why the control region could not be recovered in A. luctuosus. It would also be helpful to suggest future work, such as including more specimens and nuclear markers.

 6.Table 1 caption says "Read-processing **and SPAdes-based whole-genome assembly** statistics," but the SPAdes step is described as generating an initial mitochondrial contig via BLASTN filtering, not a full nuclear whole-genome assembly summary, clarify the caption wording so it doesn't imply whole-nuclear-genome assembly stats are reported.

Are the datasets clearly presented in a usable and accessible format, and the assembly and annotation available in an appropriate subject-specific repository?

Yes

Are sufficient details of the sequencing and extraction, software used, and materials provided to allow replication by others?

Yes

Are the rationale for sequencing the genome and the species significance clearly described?

Yes

Are the protocols appropriate and is the work technically sound?

Yes

Reviewer Expertise:

Population Genetics and Genomics, Molecular Biology, Entomology

I confirm that I have read this submission and believe that I have an appropriate level of expertise to confirm that it is of an acceptable scientific standard, however I have significant reservations, as outlined above.

# Reviewer report: Reviewer response for version 2

**DOI:** 10.5256/f1000research.208114.r522072

**Published:** 2026-09-22

**Authors:** Feiyun Tu

**Affiliations:** 1 Hainan Normal University, Haikou, Hainan, China

This study presents mitochondrial genome sequences for four species and reconstructs a phylogenetic tree based on concatenated mitochondrial protein-coding genes, successfully recovering the phylogenetic relationships among the four species. These findings provide a valuable resource for future taxonomic and phylogenetic research. The methodology is clearly described, and the authors have adequately addressed all comments raised by the previous reviewers. Therefore, I recommend that the manuscript be accepted for indexing.

Are the datasets clearly presented in a usable and accessible format, and the assembly and annotation available in an appropriate subject-specific repository?

Yes

Are sufficient details of the sequencing and extraction, software used, and materials provided to allow replication by others?

Yes

Are the rationale for sequencing the genome and the species significance clearly described?

Yes

Are the protocols appropriate and is the work technically sound?

Yes

Reviewer Expertise:

I focus on mammalian taxonomy and systematics, and phylogeography.

I confirm that I have read this submission and believe that I have an appropriate level of expertise to confirm that it is of an acceptable scientific standard.

# Reviewer report: Reviewer response for version 2

**DOI:** 10.5256/f1000research.208114.r514759

**Published:** 2026-09-21

**Authors:** Carlos Eduardo Almeida

**Affiliations:** 1 Universidade Federal de São João del Rei, Campus Centro-Oeste “Dona Lindu”, Divinópolis, Brazil

I have assessed the revised version and consider that the authors have adequately addressed the concerns raised in my previous report. I therefore recommend the manuscript for indexing and would like to amend my status from “Approved with reservations” to “Approved.”

Are the datasets clearly presented in a usable and accessible format, and the assembly and annotation available in an appropriate subject-specific repository?

Partly

Are sufficient details of the sequencing and extraction, software used, and materials provided to allow replication by others?

Yes

Are the rationale for sequencing the genome and the species significance clearly described?

Partly

Are the protocols appropriate and is the work technically sound?

Yes

Reviewer Expertise:

NA

I confirm that I have read this submission and believe that I have an appropriate level of expertise to confirm that it is of an acceptable scientific standard.

# Reviewer report: Reviewer response for version 1

**DOI:** 10.5256/f1000research.198144.r494392

**Published:** 2026-07-17

**Authors:** Carlos Eduardo Almeida

**Affiliations:** 1 Universidade Federal de São João del Rei, Campus Centro-Oeste “Dona Lindu”, Divinópolis, Brazil

This Genome Note provides useful and novel mitogenomic resources for Apiomerus, and the sampling, sequencing, annotation, data deposition and phylogenetic procedures are generally appropriate and sufficiently described. However, a few points should be addressed before full approval. Most importantly, the phylogenetic relationships reported in the Abstract are inconsistent with those described in the Results, particularly regarding the positions of A. luctuosus and A. nitidicollis, and must be reconciled with Figure 1c. The authors should also clarify the evidence supporting the designation of all four assemblies as complete, especially for Apiomerus sp., in which the boundaries of rrnL, rrnS and trnV remain unresolved, and provide mitochondrial read-depth and assembly-circularization or read-mapping evidence if available. Finally, the reported “230/260” purity ratio appears to be a typographical error for “260/230”. Subject to these focused corrections, the article will constitute a valuable and reusable genomic resource.

Are the datasets clearly presented in a usable and accessible format, and the assembly and annotation available in an appropriate subject-specific repository?

Partly

Are sufficient details of the sequencing and extraction, software used, and materials provided to allow replication by others?

Yes

Are the rationale for sequencing the genome and the species significance clearly described?

Partly

Are the protocols appropriate and is the work technically sound?

Yes

Reviewer Expertise:

Molecular ecology

I confirm that I have read this submission and believe that I have an appropriate level of expertise to confirm that it is of an acceptable scientific standard, however I have significant reservations, as outlined above.

# Response

**Published:** 2026-08-05

**Authors:** Andrés Gómez-Palacio

**Affiliations:** Universidad Pedagogica y Tecnologica de Colombia, Tunja, Boyaca, Colombia

Dr. Carlos Eduardo Almeida

*We sincerely thank you for the careful evaluation of our manuscript and for identifying important issues concerning phylogenetic consistency and the evidence supporting assembly completeness. Each comment and concern is addressed individually in the point-by-point responses below.*

 This Genome Note provides useful and novel mitogenomic resources for Apiomerus, and the sampling, sequencing, annotation, data deposition and phylogenetic procedures are generally appropriate and sufficiently described. However, a few points should be addressed before full approval.

 Most importantly, the phylogenetic relationships reported in the Abstract are inconsistent with those described in the Results, particularly regarding the positions of A. luctuosus and A. nitidicollis, and must be reconciled with Figure 1c.

 The authors should also clarify the evidence supporting the designation of all four assemblies as complete, especially for Apiomerus sp., in which the boundaries of rrnL, rrnS and trnV remain unresolved, and provide mitochondrial read-depth and assembly-circularization or read-mapping evidence if available.

***Response:*** *We thank the reviewer. We reconciled the phylogenetic relationships across the Abstract, Results, and Fig. 2c. We also reassembled and validated the mitogenomes with GetOrganelle. Apiomerus sp., A. ochropterus, and A. nitidicollis were recovered as complete circular mitogenomes, whereas A. luctuosus remains a near-complete non-circular scaffold with an incompletely recovered control region. The previous rrnL, rrnS, and trnV uncertainties in Apiomerus sp. were resolved, and read-depth, mapping-continuity, and circular-junction support are now reported.*

 Finally, the reported “230/260” purity ratio appears to be a typographical error for “260/230”. Subject to these focused corrections, the article will constitute a valuable and reusable genomic resource.

***Response:*** *We thank the reviewer for identifying this typographical error. The ratio has been corrected from “230/260” to “260/230” in the Methods. We also appreciate the reviewer’s positive assessment of the value and reusability of the genomic resource.*

# Reviewer report: Reviewer response for version 1

**DOI:** 10.5256/f1000research.198144.r494389

**Published:** 2026-07-16

**Authors:** Rui Alves

**Affiliations:** 1 University of Lleida, Lleida, Spain

This manuscript presents the first mitochondrial genomes for four *Apiomerus* species and makes these data publicly available. The specimens are vouchered, the data are deposited in GenBank and the SRA, and the phylogenetic analysis broadly supports previous relationships within Harpactorinae. These are valuable contributions.

 Still, my "partial" score has to do with the fact that the workflow from shallow whole-genome sequencing to "complete mitochondrial genomes" is insufficiently documented, and several standard assembly validation steps are absent. Most concerns can be addressed through clarification, but the current description does not allow the work to be fully reproduced or independently evaluated. The authors state that reads were assembled with SPAdes, mitochondrial contigs were identified by BLAST against *Agriosphodrus dohrni*, and "used to reconstruct complete mitochondrial genomes." Nowhere is the reconstruction process actually described. Also, a more complete explicit description of the results would help. Was each mitogenome recovered as a single circular contig? If not, how many mitochondrial contigs were obtained? How were contigs ordered? Were overlaps used? Were reference-guided joins employed? Was manual editing performed? Were gaps present? Were ambiguous bases introduced? One assembly is only **13.6 kb**, way, shorter than the others. Comments on this? Is the control region missing?  Were you able to circularize? You should also report on statistics for mapping reads back to the assembly, average and minimum read depth, show coverage plots, systematically identify of poorly supported regions. Also, did you plish the genome? Given that you are deviating from what are now standard practices, it would be nice to compare your assembly to one of the following: GetOrganelle, MitoZ, NOVOPlasty, or MitoFinder!

 Coverage estimates are also unclear and hard to interpret and compare between nuclear and mitogenomes. Please also provide, N50, longest contig, mitochondrial contig length, etc. What was the final partitioning scheme and substitution models in IQ Tree? Figure 1 displays bootstrap values. Were SH-aLRT values omitted intentionally?

Are the datasets clearly presented in a usable and accessible format, and the assembly and annotation available in an appropriate subject-specific repository?

Yes

Are sufficient details of the sequencing and extraction, software used, and materials provided to allow replication by others?

Partly

Are the rationale for sequencing the genome and the species significance clearly described?

Yes

Are the protocols appropriate and is the work technically sound?

Partly

Reviewer Expertise:

Genomics, Systems Biology, Synthetic Biology, Computational Biology

I confirm that I have read this submission and believe that I have an appropriate level of expertise to confirm that it is of an acceptable scientific standard, however I have significant reservations, as outlined above.

# Response

**Published:** 2026-08-05

**Authors:** Andrés Gómez-Palacio

**Affiliations:** Universidad Pedagogica y Tecnologica de Colombia, Tunja, Boyaca, Colombia

Dr. Rui Alves

*We sincerely thank you for the detailed and constructive assessment of our manuscript, particularly the recommendations concerning assembly reproducibility, validation, and reporting. Each comment and concern is addressed individually in the point-by-point responses below.*

 This manuscript presents the first mitochondrial genomes for four Apiomerus species and makes these data publicly available. The specimens are vouchered, the data are deposited in GenBank and the SRA, and the phylogenetic analysis broadly supports previous relationships within Harpactorinae. These are valuable contributions.

***Response:*** *We thank the reviewer for recognizing the value of the vouchered specimens, public data deposition, and phylogenetic contribution of this study.*

 Still, my "partial" score has to do with the fact that the workflow from shallow whole-genome sequencing to "complete mitochondrial genomes" is insufficiently documented, and several standard assembly validation steps are absent. Most concerns can be addressed through clarification, but the current description does not allow the work to be fully reproduced or independently evaluated. The authors state that reads were assembled with SPAdes, mitochondrial contigs were identified by BLAST against Agriosphodrus dohrni, and "used to reconstruct complete mitochondrial genomes." Nowhere is the reconstruction process actually described.

***Response:*** *We agree that the original reconstruction workflow was insufficiently documented. The Methods now describe the complete progression from filtered reads to the selected assemblies, including GetOrganelle reassembly, assembly-graph inspection, comparison with the original SPAdes/BLASTN reconstructions, read mapping, coverage and variant assessment, and circular-junction validation. The revised manuscript now classifies three assemblies as complete circular mitogenomes and A. luctuosus as a near-complete non-circularized scaffold.*

 Also, a more complete explicit description of the results would help. Was each mitogenome recovered as a single circular contig? If not, how many mitochondrial contigs were obtained? How were contigs ordered? Were overlaps used? Were reference-guided joins employed? Was manual editing performed? Were gaps present? Were ambiguous bases introduced? One assembly is only 13.6 kb, way, shorter than the others. Comments on this? Is the control region missing?  Were you able to circularize? You should also report on statistics for mapping reads back to the assembly, average and minimum read depth, show coverage plots, systematically identify of poorly supported regions. Also, did you polish the genome? Given that you are deviating from what are now standard practices, it would be nice to compare your assembly to one of the following: GetOrganelle, MitoZ, NOVOPlasty, or MitoFinder!

***Response:*** *We thank the reviewer for these detailed suggestions. We reassembled all samples with GetOrganelle and expanded the Methods and Results accordingly. A. ochropterus, A. nitidicollis, and Apiomerus sp. were recovered as single circular assemblies, whereas A. luctuosus remained a single gap-free, non-circularized scaffold with an incompletely recovered control region. No reference-guided joins, manual gap filling, gaps, or ambiguous bases were introduced. The final assemblies were validated by read remapping, coverage and variant analyses, and circular-junction support; these results and coverage summaries are now presented in Fig. 1. The read evidence did not indicate a need for additional polishing.*

 Coverage estimates are also unclear and hard to interpret and compare between nuclear and mitogenomes. Please also provide, N50, longest contig, mitochondrial contig length, etc. What was the final partitioning scheme and substitution models in IQ Tree? Figure 1 displays bootstrap values. Were SH-aLRT values omitted intentionally?

***Response:*** *We thank the reviewer. Coverage terminology and assembly statistics were clarified in Tables 1 and 2. The Methods now specify the 13 initial PCG partitions, partitioned model selection under MFP+MERGE, 1,000 ultrafast bootstrap replicates, and 1,000 SH-aLRT replicates. SH-aLRT values were calculated but intentionally omitted from Fig. 2c; the main figure displays only ultrafast-bootstrap support categories for clarity.*

# Reviewer report: Reviewer response for version 1

**DOI:** 10.5256/f1000research.198144.r494385

**Published:** 2026-07-08

**Authors:** Annabel Whibley

**Affiliations:** 1 The University of Auckland, Auckland, Auckland, New Zealand

Gómez-Palacio and colleagues report 4 complete mitochondrial genome assemblies of “bee-killer” assassin bugs.

 These assemblies were generated from vouchered specimens and low-coverage Illumina datasets. Contigs with homology to characterized mitogenomes were extracted from these assemblies and used to generate complete mitogenome representations, which were subsequently annotated. These assemblies show the expected complement of annotated genes, tRNA and rRNAs (with some ambiguity noted in one species). The assemblies are used to support a phylogenetic assessment, with additional species from public databases,

 The subdivision of the abstract into a background, methods and results section seems unusual- should this be written as a conventional narrative paragraph?

 The paper is well written and methods are sound, with a good level of background information to provide context to the work.

 The one step  that I feel is not well explained (and reason for the “partly” answers to questions on method sound-ness and reproducibility)  is the progression from the identification of contigs with mitochondrial homology to the “complete” genomes. How many, if any, of these contigs were complete assemblies? I would guess quite possibly none, but if they did obtain single contig mitochondria from the assembly this should be stated.  And if multiple contigs, how many? And what approach did the authors take to gap-filling. Ideally the location of contig breaks would be indicated on the synteny figures. Although synteny is well-conserved in this study, interpretation of the differences in the Apiomerus  sp. accession, which one would assume are derived, would be perhaps be modulated by contig breaks in the vicinity of those differences.

 Did the authors consider mapping the WGS data back to the assembled genomes? I ask in case there was any evidence of heteroplasmy and/or whether this might have resolved some of the ambiguity in the Apiomerus sp. accession. It may also have revealed any need for polishing of the resulting assemblies.

**Minor comments**

 I suggest not including the “Hahn” taxonomic in the abstract. Also, it is my understanding that “bee-assassin” is more commonly used than “bee-killer”.

 “single-locus markers such as cox1 and 16S rRNA…”.  COI might be a more familiar way to express cox1 given its extensive use in barcoding. I see that you acknowledge naming alternatives for many of these markers later.

 Can a key be included for the bootstrap significance symbols in Figure 1? And/or include qualifiers for “high” and “moderate” in the final paragraph of the text.

Are the datasets clearly presented in a usable and accessible format, and the assembly and annotation available in an appropriate subject-specific repository?

Yes

Are sufficient details of the sequencing and extraction, software used, and materials provided to allow replication by others?

Partly

Are the rationale for sequencing the genome and the species significance clearly described?

Yes

Are the protocols appropriate and is the work technically sound?

Partly

Reviewer Expertise:

Genomics, Bioinformatics

I confirm that I have read this submission and believe that I have an appropriate level of expertise to confirm that it is of an acceptable scientific standard, however I have significant reservations, as outlined above.

# Response

**Published:** 2026-08-05

**Authors:** Andrés Gómez-Palacio

**Affiliations:** Universidad Pedagogica y Tecnologica de Colombia, Tunja, Boyaca, Colombia

Dr. Annabel Whibley

*We sincerely thank you for the careful and constructive evaluation of our manuscript and for the helpful recommendations regarding its presentation and methodological clarity. Each comment and concern is addressed individually in the point-by-point responses below.*

 Gómez-Palacio and colleagues report 4 complete mitochondrial genome assemblies of “bee-killer” assassin bugs. These assemblies were generated from vouchered specimens and low-coverage Illumina datasets. Contigs with homology to characterized mitogenomes were extracted from these assemblies and used to generate complete mitogenome representations, which were subsequently annotated. These assemblies show the expected complement of annotated genes, tRNA and rRNAs (with some ambiguity noted in one species). The assemblies are used to support a phylogenetic assessment, with additional species from public databases, The subdivision of the abstract into a background, methods and results section seems unusual- should this be written as a conventional narrative paragraph?

***Response:*** *We thank the reviewer for this suggestion. The abstract has been rewritten as a single narrative paragraph without section headings. We also revised the abstract and Methods to reflect the updated GetOrganelle-based assembly workflow and the conservative classification of the resulting mitochondrial assemblies.*

 The paper is well written and methods are sound, with a good level of background information to provide context to the work.

***Response:*** *We thank the reviewer for this positive assessment of the manuscript and its methodological and contextual foundation.*

 The one step that I feel is not well explained (and reason for the “partly” answers to questions on method sound-ness and reproducibility) is the progression from the identification of contigs with mitochondrial homology to the “complete” genomes.

***Response:*** *We thank the reviewer for identifying this lack of methodological detail. The Methods section has been substantially expanded to explain the progression from mitochondrial-contig identification to the final selected assemblies. The revised workflow now describes organelle-specific reassembly with GetOrganelle, inspection of assembly graphs, comparison with the original SPAdes/BLASTN reconstructions, reciprocal sequence comparisons, read-mapping validation, and circular-junction assessment. We also revised the title and adopted conservative assembly classifications: three sequences are now described as complete circular mitogenomes, whereas A. luctuosus is described as a near-complete, non-circularized mitochondrial scaffold with an incompletely recovered control region.*

 How many, if any, of these contigs were complete assemblies? I would guess quite possibly none, but if they did obtain single contig mitochondria from the assembly this should be stated.  And if multiple contigs, how many?

***Response:*** *We thank the reviewer for this question. The original SPAdes/BLASTN analysis did not by itself establish complete mitochondrial genomes and was therefore retained only for assembly comparison. In the revised GetOrganelle analysis, one mitochondrial sequence was recovered per sample: three single circular assemblies (Apiomerus sp., A. ochropterus, and A. nitidicollis) and one single non-circular scaffold (A. luctuosus). This distinction is now stated explicitly in the Methods and Results.*

 And what approach did the authors take to gap-filling. Ideally the location of contig breaks would be indicated on the synteny figures.

***Response:*** *No manual gap filling or insertion of ambiguous bases was performed. Final sequences were recovered from GetOrganelle assembly paths and evaluated using the assembly graph and read support. Because the original SPAdes contigs were retained only for comparison, their breakpoints are not shown in the final synteny figures. The unresolved termini of the non-circular A. luctuosus scaffold are now indicated explicitly.*

 Although synteny is well-conserved in this study, interpretation of the differences in the *Apiomerus*  sp. accession, which one would assume are derived, would be perhaps be modulated by contig breaks in the vicinity of those differences.

***Response:*** *We thank the reviewer for this important observation. The original Apiomerus sp. reconstruction was indeed affected by assembly discontinuities near the region showing the apparent differences. We therefore reassembled the mitochondrial genome using GetOrganelle and validated the selected sequence through assembly-graph inspection and read remapping. The revised Apiomerus sp. mitogenome was recovered as a complete circular sequence with continuous coverage across the circular junction and the full complement of 13 protein-coding genes, 22 tRNAs, and two rRNAs. After reannotation and manual curation, the previously inferred differences were no longer supported. The four Apiomerus assemblies now show complete conservation of gene content, gene order, and transcriptional orientation, with no evidence of derived rearrangements in Apiomerus sp.*

 Did the authors consider mapping the WGS data back to the assembled genomes? I ask in case there was any evidence of heteroplasmy and/or whether this might have resolved some of the ambiguity in the Apiomerus sp. accession. It may also have revealed any need for polishing of the resulting assemblies.

***Response*** *: We thank the reviewer for this suggestion. We mapped the original paired-end reads to both assembly versions and evaluated coverage, properly paired reads, alignment errors, and high-confidence read–assembly differences. The results supported the selected GetOrganelle assemblies, resolved the previous ambiguity in Apiomerus sp., and did not indicate heteroplasmy or a need for further sequence polishing.*

 Minor comments

 I suggest not including the “Hahn” taxonomic in the abstract. Also, it is my understanding that “bee-assassin” is more commonly used than “bee-killer”.

**Response:**

*We thank the reviewer for this suggestion. We removed the taxonomic authority “Hahn” from the Abstract and replaced “bee-killer” with the more commonly used term “bee-assassin.”*

 “single-locus markers such as cox1 and 16S rRNA…”.  COI might be a more familiar way to express cox1 given its extensive use in barcoding. I see that you acknowledge naming alternatives for many of these markers later.

***Response:*** *We thank the reviewer for the suggestion. We now introduce the marker as “COI (cox1)” at first mention, while retaining cox1 in annotation-focused Methods and figures for consistency with standardized mitochondrial gene nomenclature.*

 Can a key be included for the bootstrap significance symbols in Figure 1? And/or include qualifiers for “high” and “moderate” in the final paragraph of the text.

***Response*** *: We thank the reviewer for this suggestion. We added a key to Fig. 2c defining bootstrap support as high (≥90%), moderate (70–89%), or low (<70%). Support is indicated by color-coded node symbols rather than numerical values to maintain figure clarity.*
